# Cognitive Performance in People at High Risk of Parkinson's Disease

**DOI:** 10.1002/alz70857_098325

**Published:** 2025-12-24

**Authors:** Md Shafiqur Rahman, Timothy Rittman, Brian D M Tom, Patrick F Chinnery

**Affiliations:** ^1^ MRC Biostatistics Unit, University of Cambridge, Cambridge, United Kingdom; ^2^ Department of Clinical Neurosciences, University of Cambridge, Cambridge, United Kingdom; ^3^ Department of Clinical Neurosciences, University of Cambridge, Cambridge, Cambridgeshire, United Kingdom; ^4^ Cambridge University Hospitals NHS Foundation Trust, Cambridge, United Kingdom; ^5^ MRC Mitochondrial Biology Unit, University of Cambridge, Cambridge, United Kingdom

## Abstract

**Background:**

Nearly half of Parkinson's disease (PD) patients are diagnosed with dementia within 10 years of their initial diagnosis. It is likely that these subgroups of patients experience symptoms of cognitive decline before the diagnosis of PD. Therefore, it is important to study trajectories of age‐related cognitive decline in individuals at risk of PD.

**Method:**

The National Institute for Health and Care Research's Genes and Cognition (G&C) study has conducted cognitive profiling across several domains (11 cognitive tests) in approximately 35,000 individuals. Nearly one‐third of these participants completed a second cognitive assessment approximately 41 months after the first. Participants provided basic demographic information at the time of recruitment to the NIHR Bioresource. Currently, genetic information for 9,878 participants is available, with the remaining participants undergoing genotyping. We will use the polygenic risk score (PRS) catalog number PGS004924 (90 genetic variants) to calculate the PRS for PD in G&C study participants. Individuals in the top 2.5^th^ percentile will be categorized as high‐risk, while the rest will be categorized as low risk for PD. Additionally, known common variants in GBA, LRRK2, MAPT and SNCA gene will be investigated.

**Result:**

Based on the available data, demographic and clinical characteristics will be compared between individuals with high and low polygenic risk for PD using parametric and/or non‐parametric tests. Cognitive test performance across the 11 tests will be compared between the high‐ and low‐polygenic‐risk groups for PD at both time points. Additionally, cognitive test performance will be investigated for common risk variants of PD.

**Conclusion:**

This study will provide insights into the trajectories of performance across various cognitive domains in individuals at high risk for PD. Identifying the cognitive domains most sensitive to decline in PD will support efforts for early detection and management.

